# Multiplex serum biomarker assessments: technical and biostatistical issues

**DOI:** 10.1186/1479-5876-9-173

**Published:** 2011-10-11

**Authors:** Lisa H Butterfield, Douglas M Potter, John M Kirkwood

**Affiliations:** 1 University of Pittsburgh Cancer Institute, 5117 Centre Avenue, suite 1.27, Pittsburgh, PA 15213, USA; 2 University of Pittsburgh School of Medicine, 5117 Centre Avenue, suite 1.27, Pittsburgh, PA 15213, USA; 3 Department of Medicine, University of Pittsburgh, 5117 Centre Avenue, suite 1.27, Pittsburgh, PA 15213, USA; 4 Department of Surgery, University of Pittsburgh, 5117 Centre Avenue, suite 1.27, Pittsburgh, PA 15213, USA; 5 Department of Immunology, University of Pittsburgh, 5117 Centre Avenue, suite 1.27, Pittsburgh, PA 15213, USA; 6 Depament of Biostatistics, University of Pittsburgh, 5117 Centre Avenue, suite 1.27, Pittsburgh, PA 15213, USA

**Keywords:** serum, biomarkers, melanoma, cytokine, Luminex

## Abstract

**Background:**

Identification of predictive and prognostic biomarkers for patients with disease and undergoing different therapeutic options is a very active area of investigation. Many of these studies seek biomarkers among circulating proteins accessed in blood. Many levels of standardization in materials and procedures have been identified which can impact the resulting data.

**Methods:**

Here, we have observed unexpected variability in levels of commonly tested analytes in serum which were processed and stored under standardized conditions. We have identified apparent changes in cytokine, chemokine and growth factor levels detected by multiplex Luminex assay in melanoma patient and healthy donor serum samples, over storage time at -80°C. Controls included Luminex kit standards, multiplexed cytokine standards and WHO cytokine controls. Data were analyzed by Wilcoxon rank-sum testing and Spearman's test for correlations.

**Results:**

The interpretation of these changes is confounded by lot-to-lot kit standard curve reagent changes made by a single manufacturer of Luminex kits.

**Conclusions:**

This study identifies previously unknown sources of variation in a commonly used biomarker assay, and suggests additional levels of controls needed for identification of true changes in circulating protein levels.

## Background

To improve the clinical efficacy of immunotherapies and our ability to stratify patients rationally for therapeutic intervention, biomarkers are critical to progress. The FDA's Critical Path prioritizes development of biomarkers, including a focus on aspects of: Biospecimens, Analytical Performance, Standardization and Harmonization and Bioinformatics. Accurate biomarkers offer the prospect for earlier diagnosis, improved precision of application of expensive and toxic therapies on the optimal patient populations, monitoring disease progression and therapeutic benefits as well as accelerating drug development and discovery. Guidelines for incorporation of biomarker studies in early clinical trials of novel agents have been published [1].

There is a critical need for development and validation of biomarkers to identify patients who can benefit from a particular form of immunotherapy. Only a fraction of patients benefit from IFN-α treatment [2], only a fraction of patients can achieve durable regressions in response to antigen vaccination [3], or antibody therapies, and we do not yet know the mechanisms responsible for therapeutic benefit. Despite substantial efforts from many groups, we do not know which parameters of immune response (and which assays used to assess these parameters) yield optimal results for efficacy analysis [4-7]. A major reason for this has been that objective clinical response rates are often below 10%, confounding the measurement of significant correlations between biomarkers and clinical responses in studies of modest size. Another important issue is that assay results may depend on biological specimen handling before assessment, and on methodological differences in complex, high throughput assays.

A number of studies in melanoma have identified candidate biomarkers of response to therapy. These range from circulating cytokines and growth factors [8,9], gene expression profiles in tumors [10], circulating tumor cells [11], serum autoantibody profiling [12] and tumor specific T cell IFN-γ production [13] to molecular signaling pathways in tumors [14] and the nature of tumor infiltrating cells [15]. The vast majority of candidate biomarkers have not yet achieved routine clinical use due to lack of reproducibility, need for new technology and equipment, need for high quality tumor samples or high cost. The relative ease of collecting, processing, storing and shipping blood has made it a common resource for biomarker testing.

Several reports have identified phenotypic and functional changes in blood cells and serum components when the blood is held for hours or days and at different temperatures before processing [16-18]. These time-dependent and temperature-dependent effects should be controlled for to the extent possible before blood processing. Standardized processing procedures by trained and competency-tested personnel can also improve immunologic assay data consistency [19]. In addition, use of freezers for sample storage that are monitored for temperature stability and that have 24 hours-a-day alarm response eliminates concerns that samples might undergo freeze-thaw cycles or be otherwise compromised by temperature changes during storage. Many of these central laboratory procedures for processing, storage and equipment maintenance are mandated by accreditation groups such as CLIA and FACT, and are described in resources from CLSI [20-22].

During an investigation of biomarkers of prolonged survival after IFN-α treatment in banked melanoma patient serum samples, we discovered a number of both technical and biostatistical analysis issues [23]. Our preliminary results identified a large number of serum cytokines that appeared to correlate significantly with survival. However, further dissection of the data revealed a number of technical issues that made interpretation of the data impossible.

Here, we have performed a time course analysis of cytokines, chemokines and growth factors measured in the banked serum of healthy donors and melanoma patients stored for various intervals, and analyzed by multiplex Luminex assay. We find that a number of these analytes appear to be unstable during storage. We have also tested several aspects of the Luminex assay performance and identified a number of concerns with these multiplexed assays. Biostatistical tests indicate that despite several layers of procedural standardization and levels of controls, reliable multiplexed cytokine and chemokine determinations may be compromised by length of time in storage and/or by the changes regularly made by assay kit manufacturers to different lots and the analyte standards included. These results raise concerns about serum biomarker studies and suggest that additional controls may be required to confidently compare levels over time and between lots of reagents from the same manufacturer.

## Methods

### Study subjects

All serum samples were obtained after written informed consent, and under IRB approved protocols of investigation at the University of Pittsburgh. The samples received in 2005 were obtained from 23 patients at two clinical sites (Pennsylvania and Indiana). The UPCI #96-099 banking protocol was utilized for the five 2010 melanoma patient sera tested. The UPCI #04-001 healthy donor blood collection protocol was used for the blood obtained from 10 healthy donors in 2010.

### Blood processing and banking

For serum collection, red top vaccutainer tubes (no anticoagulant) provided by our laboratory (Becton Dickinson #6430) in kits were used. Upon arrival in the lab, the samples are checked for proper identification, given accession numbers, and either processed immediately or (if received after 4 pm) put in the refrigerator (at 4°C) for processing the next morning. All samples were processed within 24 hours, including those drawn at external sites and shipped at ambient temperature overnight in insulated shipping containers. All processing was performed by technologists who received the same training, and the laboratory SOP #0108 was followed. Technologists also undergo annual competency training. Samples were centrifuged for 10 min at 2, 500 rpm in a refrigerated centrifuge at 4°C, then the serum was aliquoted into polypropylene freezer vials at 1.1 mL per vial and immediately placed in a -80°C freezer. All samples were stored in a monitored freezer until testing, freezer temperatures did not fluctuate above -55°C (during brief periods of high use). Samples were thawed before testing and repeated testing was performed on separate aliquots to eliminate variability from freeze-thaw cycles. The laboratory is certified under the Pennsylvania Department of Health, College of American Pathologists (CAP) and Clinical Laboratory Improvement Amendments (CLIA for Histocompatibility and General Immunology). The laboratory is registered with the FDA, and maintains a facilities master file (BB-MF-12244). The exploratory Luminex assay reported here is not used for clinical decision making, and is not a CLIA-certified assay.

### Luminex assay and controls

The Luminex kits were obtained from the same manufacturer, which changed ownership during the period of the study (BioSource, Invitrogen, Life Technologies). Assays were performed only on serum samples that had been stored at -80°C. Serum samples were thawed in a refrigerator overnight (healthy donor controls, < 12 hours total time) or at room temperature the day of the assay (patient samples), clarified in a microfuge for 10 min at 1, 000 g, then diluted with the assay diluent provided per assay manufacturer's instructions. Healthy donor and control samples were run in duplicate, but large numbers of patient sera were run in singlets. The same trained technologist performed all of the assays reported herein, according to the same laboratory SOP #0037). The software used for all assays was the BioPlex System BioPlex Manager 4.0, which uses 5-parameter logistic regression. Each sample acquired ≥ 100 bead events, per manufacturers' instructions. Analytical sensitivity was calculated based on two standard deviations from the background MFI of the standard curve. There were no changes in the antibodies used for the analytes of interest reported here, and the standards were benchmarked in the same way over the time period tested here. R&D QC controls (R&D Systems QC02) are reconstituted with assay diluent from the Hu Extracellular buffer kit LHB0001 (BioSource). Each lot provides expected values for several commonly tested cytokines (as measured by R&D Systems ELISA assays). Additional kit details are presented in Additional File 1, Table S1.

To address potential inter-analysis variability, 770 data points from 2005 and 430 data points from 2010 were re-analyzed at the same time (2011) with version 6.0 software, on the original machine. There were 0/1, 200 changes in the resulting absolute values obtained.

### WHO cytokine standards

WHO cytokine standards were resuspended as follows: 117187 GM-CSF WHO 88/646 10, 000 IU: contents of the ampoule were dissolved with 0.5 mL sterile distilled water and brought up to 1 mL with PBS. Further 1:10 dilution was performed with AIM V (Invitrogen) medium. 117173 IL-4 WHO 88/656 0.1 μg = 1, 000 arbitrary units per ampoule: contents of the ampule were resuspended with PBS/1% BSA, and the 1:10 dilution was performed with AIM V. 117184 IL-10 WHO 92/516 1 μg = 5, 000 RU per ampoule: contents of the ampoule were dissolved with 0.5 mL sterile distilled water and then brought up to 1 mL with PBS. Further 1:10 dilution was performed with AIM V. 117177 IL-8 WHO 89/520 1 μg = 1, 000 RU per ampoule = 1, 000, 000 pg/mL: contents of the ampoule were resuspended with PBS/1% BSA and the 1:10 dilution was performed with AIM V. To assay the WHO standards, each was diluted 1:10 (20 μL WHO standard dilution (above) + 180 μL assay diluent) and 1:50 (10 μL WHO + 490 μL assay diluent). The dilutions were treated as samples in the assay, such that the final dilutions were 1:20 and 1:100, relative to the Luminex kit standard curve (the assayed well contains 50 μL of the dilution + 50 μL of assay diluent).

### Biostatistical Methods

Analyte concentrations were compared at two time points with a one-sample Wilcoxon rank-sum test on the ratio of the two concentrations. Correlation was assessed with Spearman's test. All p-values are two-sided. Assay results below the lower limit of detection or above the upper limit of quantitation were not used in the analysis.

## Results and Discussion

During the analysis of a retrospective biomarker study conducted with a set of banked sera from melanoma patients [23], we discovered a potential correlation between the levels of analytes measured by Luminex and the time that the sera were stored at -80°C. Therefore, we examined several aspects of serum storage and the Luminex assay.

### Repeat testing in 2010 of sera stored in 2005

Our first sample set consisted of 23 melanoma patient sera (the "old patients") who had a blood sample drawn in 2005, and had a Luminex assay performed on serum samples, on either 10/31/2005, 11/01/2005 or 2/17/2006; we refer to these as the "early" assays. To determine any changes over storage time, we thawed aliquots (not previously thawed) and tested a subset of the analytes originally tested, again by Luminex (Table 1). Unexpectedly, we identified a number of apparent changes in analyte levels. We repeated these measurements up to three times (depending on the number of previously untouched aliquots remaining) for these 23 samples: (2/02/10, 5/13/2010 and 8/11/2010)--the "late" assays. Seven of the 10 analytes we examined had highly significant changes during the approximately 5 years of storage at -80°C.

**Table 1:** Old patient Serum Samples

Sample	Date Drawn	Draw Date	Assay Date	IL-4 pg/mL	IL-6 pg/mL	IL-8 pg/mL	IL-10 pg/mL	TNF-α pg/mL	IFN-g pg/mL	GM-CSF pg/mL	IP-10 pg/mL	MIG pg/mL	MCP-1 pg/mL
**patient 1**	6/2/2005	6/1/2005	10/31/2005	< 5	217	64	< 10	38	29	< 15	1339	62	10145
			8/11/2010	25	106	353	6	< 10	28	< 15	1214	48	> 7200
			8/11/2010	16	98	370	6	< 10	29	< 15	1195	42	> 7200
													
**patient 2**	6/2/2005	6/1/2005	10/31/2005	< 5	41	24	< 10	19	23	< 15	> 2800	130	2725
			8/11/2010	13	20	132	7	< 10	24	< 15	> 9600	164	3149
			8/11/2010	13	22	144	7	< 10	26	< 15	> 9600	162	2989
													
**patient 3**	6/2/2005	6/1/2005	10/31/2005	< 5	13	23	< 10	7	< 14	< 15	55	62	384
			8/11/2010	5	5	125	5	< 10	< 5	< 15	83	90	419
			8/11/2010	7	6	151	5	< 10	< 5	< 15	83	98	455
													
**patient 4**	7/30/2005	7/30/2005	11/1/2005	32	49	17	17	83	95	173	64	241	394
			2/2/2010	47	17	75	20	21	63	132	87	209	633
			5/13/2010	42	14	73	20	20	51	124	84	153	554
			5/13/2010	42	15	69	22	21	62	133	84	154	518
			8/11/2010	55	21	89	27	28	118	89	90	213	140
			8/11/2010	47	23	87	33	32	144	105	90	209	128
													
**patient 5**	8/9/2005	8/9/2005	11/1/2005	12	2199	266	43	100	178	< 15	> 2800	407	> 17800
			2/2/2010	6	1105	1469	< 14	17	159	37	2178	374	11991
			5/13/2010	13	949	1494	< 14	16	140	36	2476	313	10275
			5/13/2010	12	971	1428	< 14	13	121	24	2233	300	9045
													
**patient 6**	8/9/2005	8/9/2005	11/1/2005	14	592	171	28	52	229	< 15	> 2800	2586	10705
			2/2/2010	10	350	1016	< 14	< 10	198	37	2176	2039	11703
			5/13/2010	13	275	971	< 14	< 10	155	18	> 2980	2276	9492
			5/13/2010	< 5	270	968	< 14	< 10	160	11	> 2980	2294	10581
													
**patient 7**	8/15/2005	8/15/2005	11/1/2005	< 5	19	47	< 5	13	< 14	< 15	285	40	2565
			5/13/2010	8	24	242	< 14	< 10	31	< 15	445	25	5453
			5/13/2010	< 5	20	234	< 14	< 10	27	< 15	360	25	5088
													
**patient 8**	8/20/2005	8/20/2005	11/1/2005	36	48	17	17	80	111	197	27	182	379
			2/2/2010	46	17	99	23	23	78	146	45	172	614
			5/13/2010	48	16	87	26	25	84	175	40	128	550
			5/13/2010	50	15	84	22	21	70	151	41	117	544
			8/11/2010	33	15	89	23	21	103	85	47	136	119
			8/11/2010	49	21	107	29	30	136	95	48	150	125
													
**patient 9**	9/15/2005	9/15/2005	11/1/2005	42	67	21	11	72	122	208	275	383	1119
			5/13/2010	77	22	78	28	36	80	224	346	328	2162
			5/13/2010	80	22	76	27	34	87	233	347	306	2270
													
**patient 10**	9/19/2005	9/19/2005	2/17/2006	< 5	19	45	< 5	13	< 7	< 15	183	42	1656
			5/13/2010	8	24	287	< 14	< 10	26	< 15	350	21	5509
			5/13/2010	8	22	290	< 14	< 10	29	< 15	347	17	5021
													
**patient 11**	9/16/2005	9/22/2005	11/1/2005	48	94	2675	24	142	135	201	902	419	10026
			5/13/2010	85	37	12663	29	39	114	272	988	300	13807
			5/13/2010	77	37	13690	27	34	114	266	1337	285	14377
			8/11/2010	93	41	14814	26	59	72	219	716	340	> 7200
			8/11/2010	110	48	12823	30	58	80	238	793	364	> 7200
**patient 12**	9/28/2005	9/28/2005	2/17/2006	62	92	27	39	82	183	328	51	270	436
			2/2/2010	46	22	92	36	20	118	222	88	200	909
			5/13/2010	43	18	75	35	20	109	221	76	125	741
			5/13/2010	34	18	88	35	22	124	208	78	165	755
													
**patient 13**	10/6/2005	10/5/2005	11/1/2005	28	67	51	8	80	77	131	877	326	6818
			8/11/2010	83	42	342	29	55	64	225	725	332	> 7200
			8/11/2010	116	44	335	33	72	79	228	802	356	7345
													
**patient 14**	10/7/2005	10/6/2005	11/1/2005	42	67	24	18	66	122	156	353	711	1266
			8/11/2010	89	39	118	35	66	59	237	314	906	1088
			8/11/2010	70	35	114	30	53	52	189	328	891	1057
													
**patient 15**	10/12/2005	---	2/17/2006	51	76	26	32	86	180	276	63	257	395
			2/2/2010	38	21	113	48	23	146	255	107	245	801
			5/13/2010	35	17	95	44	23	116	205	89	170	601
			5/13/2010	28	15	100	42	20	107	224	82	168	576
													
**patient 16**	10/17/2005	---	2/17/2006	< 5	54	60	< 5	34	< 7	< 15	434	55	3950
			5/13/2010	5	41	368	< 14	< 10	39	< 15	780	25	10159
			5/13/2010	11	43	373	< 14	< 10	39	< 15	846	23	10552
			8/11/2010	21	52	402	6	< 10	6	15	464	35	4616
			8/11/2010	8	29	391	< 5	< 10	< 5	< 15	465	35	4777
													
**patient 17**	11/3/2005	11/3/2005	2/17/2006	23	52	11	< 5	49	75	111	155	283	686
			5/13/2010	72	23	57	24	31	67	221	198	288	1734
			5/13/2010	17	9	27	< 14	< 10	14	53	186	218	1706
			8/11/2010	90	36	63	28	60	67	222	203	370	757
			8/11/2010	85	30	60	24	52	56	213	202	325	705
													
**patient 18**	11/16/2005	--	2/17/2006	7	18	14	< 5	20	29	44	41	79	327
			2/2/2010	16	15	74	< 14	< 10	34	51	59	93	807
			5/13/2010	10	12	71	< 14	< 10	20	24	57	50	611
			5/13/2010	8	10	72	< 14	< 10	24	30	52	48	617
													
**patient 19**	11/16/2005	---	2/17/2006	10	48	18	< 5	41	39	67	144	96	1143
			2/2/2010	16	30	116	< 14	< 10	43	75	248	108	2812
			5/13/2010	13	22	100	< 14	10	35	67	202	75	2101
			5/13/2010	11	24	108	< 14	< 10	38	61	220	67	2304
													
**patient 20**	12/8/2005	12/8/2005	2/17/2006	27	31	10	< 5	62	73	149	35	162	452
			2/2/2010	49	17	61	24	24	89	146	60	162	1422
			5/13/2010	43	12	48	19	24	70	137	52	108	1114
			5/13/2010	41	12	50	21	21	77	137	55	106	1234
													
**patient 21**	12/12/2005	12/9/2005	2/17/2006	< 5	24	41	< 5	16	< 7	< 15	537	35	1236
			5/13/2010	8	22	235	< 14	< 10	29	< 15	682	25	3153
			5/13/2010	10	23	233	< 14	< 10	27	< 15	789	25	3457
			8/11/2010	14	29	235	11	< 10	7	< 15	518	34	1423
			8/11/2010	13	25	238	11	< 10	6	< 15	546	39	1322
													
**patient 22**	1/26/2006	1/25/2006	2/17/2006	8	30	4	< 5	24	24	40	706	216	24
			8/11/2010	47	22	44	17	45	37	196	332	283	888
			8/11/2010	56	26	47	19	53	42	223	318	283	959
													
**patient 23**	1/26/2006	1/25/2006	2/17/2006	8	56	68	< 5	75	20	< 15	8705	266	75
			8/11/2010	75	3953	534	19	56	50	202	650	339	> 7200
			8/11/2010	76	4542	525	19	58	48	210	695	318	> 7200
					223								

**Figure 1:**
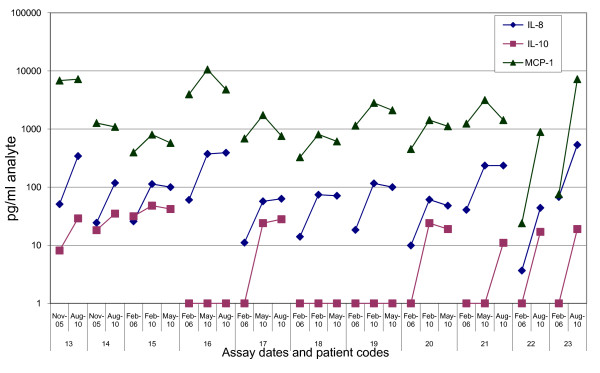
**Representative cytokine and chemokine changes over time**. Data are shown for old patients 13 through 23, for cytokines IL-8 and IL-10, and chemokine MCP-1. On a log scale, changes detected between 2005-2006 and 2010 assays are shown.

There were different patterns seen for different groups of analytes, some of which were relatively stable over time (IL-4, change over time: p = 0.28) while others were found to change (IL-10, p = 0.093; GM-CSF, p = 0.11). Levels of some of the analytes decreased over the storage time (IL-6, p = 0.00021; decreasing in 21/23 samples; TNFα, p = 0.0078, decreasing in 20/23). Surprisingly, the IL-8 levels were significantly increased from the initial test to the subsequent tests 5 years later (IL-8, p = 0.000030, approximately 5-fold increased in 23/23 patient samples). MCP-1 levels also increased in a majority of samples (MCP-1, p = 0.00012) (Table 1/Figure 1). Each p-value was computed with a one-sample Wilcoxon test on the ratio of the 5/13/2010 assay result (for which we had the most data) to the result of the early assay.

### Healthy donor and melanoma patient serum time course in 2010

To determine whether we could detect similar changes over a period of months, we drew blood from 10 healthy donors (HD, Additional File 2, Table S2, Table 2 data) and 5 melanoma patients ("new patients") (Additional File 3, Table S3, Table 3 data). HD samples were tested initially 2 months after processing and freezing, and then twice more, at 5 and 8 months of storage on the same dates as the old patient sample described above. The melanoma patient samples were tested 2 days after processing and cryopreservation, and again 3 months later.

**Table 2:** Healthy Donor Sera Analysis

Sample	Date Drawn	Assay Date	IL-4 pg/mL	IL-6 pg/mL	IL-8 pg/mL	IL-10 pg/mL	TNF-α pg/mL	IFN-g pg/mL	GM-CSF pg/mL	IP-10 pg/mL	MIG pg/mL	MCP-1 pg/mL
**Healthy donor 1**	12/14/2009	2/2/2010	57	43	25	21	16	44	90	28	143	324
		2/2/2010	58	48	22	25	16	49	94	30	143	319
		5/13/2010	50	39	19	22	13	31	87	25	103	263
		5/13/2010	52	29	18	17	11	29	78	25	100	254
		8/11/2010	36	42	11	25	< 10	20	40	31	71	30
		8/11/2010	29	39	11	20	< 10	16	39	29	64	31
												
**Healthy donor 2**	12/16/2009	2/2/2010	57	61	44	41	20	160	179	31	96	632
		2/2/2010	59	59	40	42	18	163	151	31	105	589
		5/13/2010	44	48	29	31	15	109	129	28	70	529
		5/13/2010	41	49	34	33	15	109	128	27	69	521
		8/11/2010	82	80	63	37	42	100	84	40	151	160
		8/11/2010	67	91	68	39	34	105	81	35	165	138
												
**Healthy donor 3**	12/17/2009	2/2/2010	17	< 8	18	< 14	< 10	< 12	< 15	23	20	977
		2/2/2010	21	< 8	19	< 14	< 10	< 12	< 15	23	13	921
		5/13/2010	22	< 8	21	< 14	< 10	< 12	< 15	22	< 12	803
		5/13/2010	20	< 8	18	< 14	< 10	< 12	< 15	23	< 12	763
		8/11/2010	24	< 3	18	< 5	< 10	< 5	< 15	19	11	241
		8/11/2010	32	< 3	21	< 5	< 10	< 5	< 15	23	16	258
												
**Healthy donor 4**	12/18/2009	2/2/2010	111	29	88	51	50	189	253	39	196	577
		2/2/2010	121	31	90	51	56	212	262	37	216	579
		5/13/2010	81	20	66	38	39	147	211	30	128	468
		5/13/2010	76	21	60	36	33	142	201	30	133	440
		8/11/2010	232	48	160	65	90	137	173	49	277	171
		8/11/2010	222	46	167	68	92	141	167	48	276	183
												
**Healthy donor 5**	12/21/2009	2/2/2010	12	9	33	< 14	< 10	18	33	20	32	194
		2/2/2010	< 5	< 8	30	< 14	< 10	14	25	20	13	192
		5/13/2010	5	< 8	34	< 14	< 10	14	< 15	19	< 12	177
		5/13/2010	< 5	< 8	32	< 14	< 10	< 12	< 15	20	12	174
		8/11/2010	7	7	26	8	< 10	> 5	< 15	25	20	16
		8/11/2010	12	12	33	10	< 10	< 5	23	27	20	17
												
**Healthy donor 6**	12/21/2009	2/2/2010	19	8	24	< 14	< 10	< 12	37	22	50	496
		2/2/2010	< 5	< 8	28	< 14	< 10	< 12	17	22	37	558
		5/13/2010	19	< 8	15	< 14	< 10	< 12	18	19	40	434
		5/13/2010	10	< 8	< 12	< 14	< 10	< 12	18	18	36	413
		8/11/2010	12	12	47	12	14	7	21	27	48	111
		8/11/2010	18	11	45	13	13	7	21	28	51	108
												
**Healthy donor 7**	12/22/2009	2/2/2010	16	< 8	38	< 14	< 10	35	56	19	66	1040
		2/2/2010	17	9	40	< 14	10	35	62	19	66	1019
		5/13/2010	19	< 8	35	< 14	< 10	40	51	17	50	843
		5/13/2010	20	< 8	41	< 14	< 10	33	51	18	53	848
		8/11/2010	13	6	28	28	< 10	19	37	21	24	245
		8/11/2010	16	10	30	41	11	25	49	24	24	246
												
**Healthy donor 8**	12/23/2009	2/2/2010	54	15	39	29	17	82	135	40	188	926
		2/2/2010	58	17	33	29	17	78	123	42	188	934
		5/13/2010	64	15	37	32	21	76	144	38	160	815
		5/13/2010	65	15	35	33	20	72	129	36	160	742
		8/11/2010	23	< 3	10	8	< 10	9	22	34	71	144
		8/11/2010	32	6	14	14	< 10	21	36	34	96	130
												
**Healthy donor 9**	12/24/2009	2/2/2010	< 5	9	17	< 14	< 10	< 12	< 15	21	13	969
		2/2/2010	< 5	8	15	< 14	< 10	< 12	< 15	20	20	928
		5/13/2010	< 5	< 8	13	< 14	< 10	< 12	< 15	17	< 12	784
		5/13/2010	< 5	< 8	14	< 14	< 10	< 12	< 15	19	< 12	813
		8/11/2010	8	11	19	9	< 10	6	< 15	29	20	332
		8/11/2010	7	10	17	8	< 10	< 5	< 15	26	20	331
												
**Healthy donor 10**	12/28/2009	2/2/2010	< 5	8	< 12	< 14	< 10	< 12	< 15	37	13	1034
		2/2/2010	< 5	< 8	< 12	< 14	< 10	16	< 15	37	13	990
		5/13/2010	< 5	< 8	< 12	< 14	< 10	< 12	< 15	34	< 12	845
		5/13/2010	< 5	< 8	< 12	< 14	< 10	< 12	< 15	36	< 12	802
		8/11/2010	< 5	4	< 3	< 5	< 10	< 5	< 15	57	6	374
		8/11/2010	5	5	< 3	< 5	< 10	8	< 15	59	11	385

**Table 3:** New Melanoma Patient Sera Analysis

Sample	Draw Date	Assay Date	IL-4 pg/mL	IL-6 pg/mL	IL-8 pg/mL	IL-10 pg/mL	TNF-α pg/mL	IFN-g pg/mL	GM-CSF pg/mL	IP-10 pg/mL	MIG pg/mL	MCP-1 pg/mL
**Mel. Pt. 1**	5/10/2010	5/13/2010	15	11	42	< 14	95	24	< 15	39	12	754
	5/10/2010	5/13/2010	10	8	39	< 14	82	22	< 15	40	17	754
	5/10/2010	8/11/2010	21	12	44	22	183	20	41	49	32	270
	5/10/2010	8/11/2010	24	13	43	29	168	23	51	50	32	268
												
**Mel. Pt. 2**	5/10/2010	5/13/2010	18	17	87	< 14	< 10	26	< 15	30	21	1437
	5/10/2010	5/13/2010	13	16	95	< 14	< 10	21	< 15	31	17	1494
	5/10/2010	8/11/2010	28	30	97	22	10	25	33	38	39	664
	5/10/2010	8/11/2010	25	31	86	24	10	24	30	37	28	662
												
**Mel. Pt. 3**	5/10/2010	5/13/2010	42	21	72	29	20	< 12	18	190	81	771
	5/10/2010	5/13/2010	38	19	70	25	17	< 12	18	188	78	732
	5/10/2010	8/11/2010	34	14	73	31	38	9	47	141	96	223
	5/10/2010	8/11/2010	36	15	70	28	32	6	50	135	89	198
												
**Mel. Pt. 4**	5/10/2010	5/13/2010	68	26	45	51	19	62	107	19	145	955
	5/10/2010	5/13/2010	66	24	42	53	18	63	111	18	143	875
	5/10/2010	8/11/2010	102	36	26	120	< 10	16	50	19	100	238
	5/10/2010	8/11/2010	99	41	24	139	< 10	16	48	20	103	215
												
**Mel. Pt. 5**	5/10/2010	5/13/2010	35	64	380	< 14	13	27	78	27	106	831
	5/10/2010	5/13/2010	34	61	393	< 14	10	27	82	26	101	737
	5/10/2010	8/11/2010	33	43	458	20	16	27	54	32	139	170
	5/10/2010	8/11/2010	45	53	480	25	24	34	55	38	146	222

**Figure 2:**
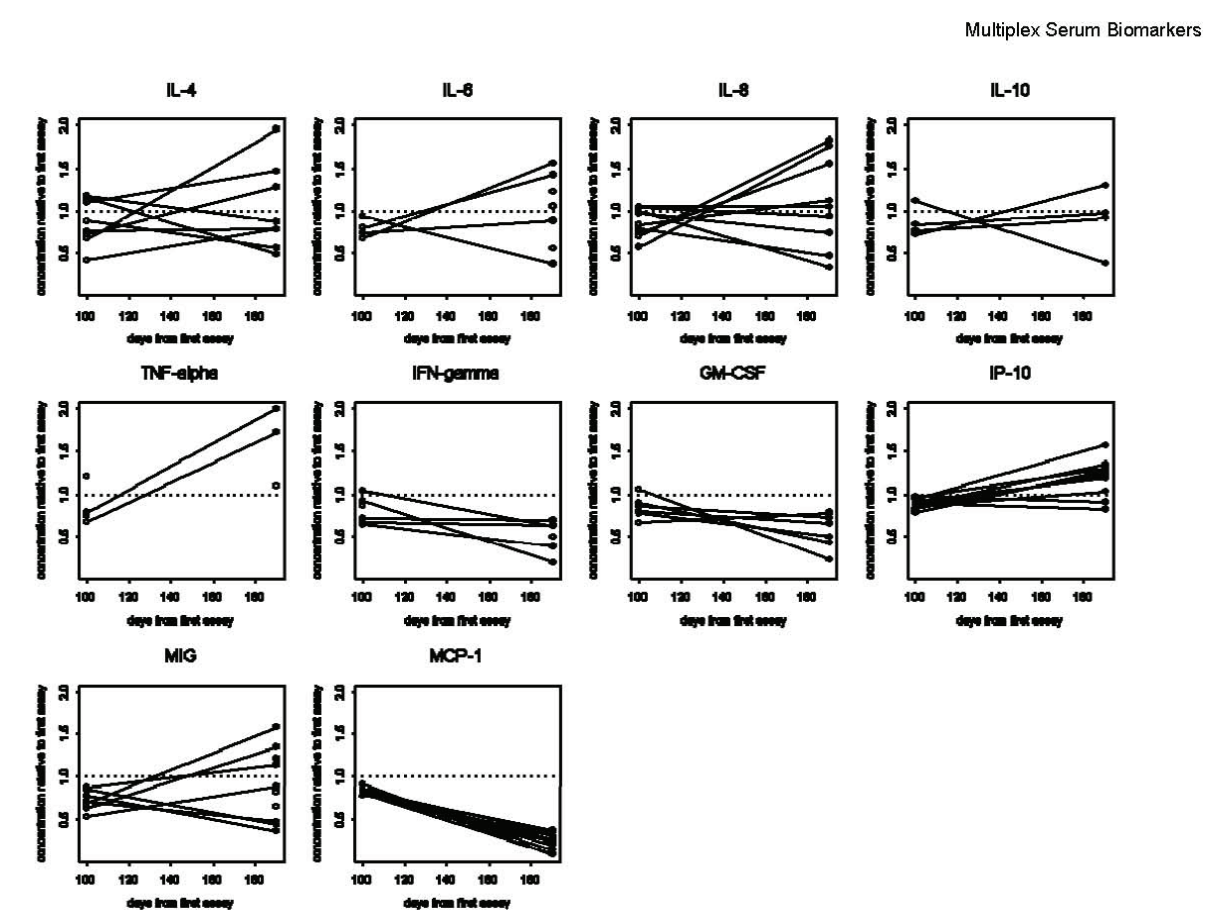
**Time course of analyte concentrations for healthy donors**. Assays done on 5/13/2010 and 8/11/2010 were normalized to those done on 2/02/2010.

**Figure 3:**
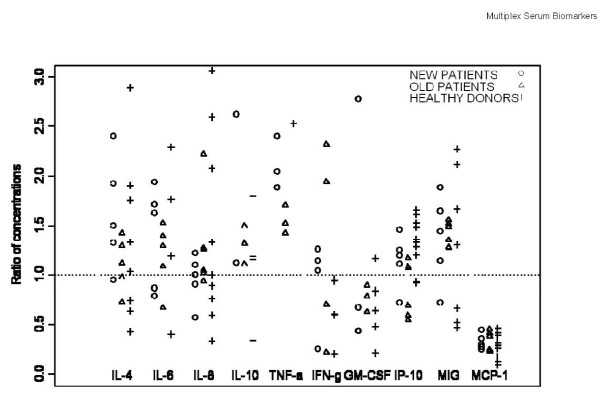
**Comparison of assay results obtained with sera from healthy donors, new melanoma patients and old melanoma patients**. Points are the ratio of concentrations of the assays done on 8/11/2010 normalized to those done on 5/13/2010.

As expected, HD samples had low circulating levels of many analytes tested. These HD control samples also showed changes in analyte levels, even after short-term storage. Again, some analytes were stable, others were much less stable. IL-8 increased in 3/10 HD, at the 8 month timepoint (n.s.), but not by 5 months. IP-10 also began to increase in 5/10 HD at 8 months (p = 0.01). Several analytes decreased in the relatively short storage time interval, including IFNγ (p = 0.06 at 5 mo., p = 0.03 at 8 mo., decreasing in 6/10 HD), and MCP-1, which showed the most dramatic decreases in 10/10 donors, by 8 mo. (p = 0.002). These changes, between the first assay and the second and third assays (100 and 190 days apart), are shown graphically in Figure 2. The melanoma patient samples did not show significant changes within the short storage time, with the exception of MCP-1, which decreased in 5/5 samples within 3 months (p = 0.06). When the ratios of the concentrations of the different analytes measured at different times were plotted together (Figure 3), the trends in concentration changes observed were not significantly different between the serum sample data sets (old patients, HD, new patients) (Table 1, Table 2, Table 3).

### Cytokine Controls used in assays

We purchased our Luminex kits from a single source, however, that source changed ownership between Oct. '05 and Aug. '10 (from Biosource to Invitrogen to Life Technologies). Each kit includes reagents to generate an 8-point standard curve from which all values are determined. For the custom kits we requested, to test a specific array of analytes of interest, the manufacturer pre-tests the specific antibodies together, to confirm lack of cross-reactivity. The manufacturer indicates that the kits are not released unless the following criteria are met: " < 10% cross-reactivity to related recombinant protein at the highest point of the standard curve" (Life Technologies). We requested the specific cross-reactivity testing data performed for the kits we used in this study, but were repeatedly informed that company policy prohibits QC data release to customers.

As an additional control, we included "Multiplex QC" controls, which are complex mixtures of recombinant cytokines, chemokines and growth factors prepared by the manufacturer at 3 concentrations (low, medium and high). We have established the reproducibility of this control (Additional File 4, Table S4) when tested via Luminex (% CV = 1%-52%, average % CV = 14% for 8 analytes). While the absolute values for each analyte do not exactly match the "expected" value from the QC control manufacturer (R&D Systems), they are similar, and we use a different platform and different antibody clones for detection via Luminex, which may account for those differences (as indicated in the package insert).

We also received WHO cytokine standards for IL-4, IL-8, IL-10 and GM-CSF. These lyophilized cytokine controls were resuspended (Materials and Methods) and individually tested at 1:10, 1:50 and 1:100 dilutions in two replicate Luminex assays for the same ten analytes described above. These data are presented in Table 4. As expected, the standard under study was almost always detected. However, there were some surprising results. MCP-1 was also almost always detected in addition to the standard, and MIG was always detected when the standard IL-10 was used. The apparent concentrations of these two analytes in some instances exceeded 10% of that of the standard. IL-6, IFN-γ and GM-CSF also showed evidence of minor cross-reactivity.

**Table 4:** WHO Cytokine Standards

Lab Number	Assay Date	IL-4 pg/mL	IL-6 pg/mL	IL-8 pg/mL	IL-10 pg/mL	TNF-α pg/mL	IFN-g pg/mL	GM-CSF pg/mL	IP-10 pg/mL	MIG pg/mL	MCP-1 pg/mL
**117173 IL-4**											
1:10	40311	17497	N/A	N/A	N/A	N/A	N/A	N/A	N/A	N/A	N/A
1:10	40401	11364	N/A	N/A	N/A	N/A	N/A	N/A	N/A	N/A	196
1:10	40401	10956	N/A	N/A	N/A	N/A	N/A	N/A	N/A	N/A	114
1:50	40311	10945	N/A	N/A	N/A	N/A	N/A	N/A	N/A	N/A	N/A
1:50	40401	1350	N/A	N/A	N/A	N/A	N/A	N/A	N/A	N/A	392
1:50	40401	1321	N/A	N/A	N/A	N/A	N/A	N/A	N/A	N/A	1220
											
**117177 IL-8**											
1:10	40311	N/A	N/A	216983	N/A	N/A	N/A	N/A	N/A	N/A	N/A
1:10	40401	N/A	N/A	153880	N/A	N/A	N/A	N/A	N/A	N/A	563
1:10	40401	N/A	N/A	153707	N/A	N/A	N/A	N/A	N/A	N/A	509
1:50	40311	N/A	N/A	QA	N/A	N/A	N/A	N/A	N/A	N/A	N/A
1:50	40401	N/A	N/A	45621	N/A	N/A	N/A	N/A	N/A	N/A	2169
1:50	40401	N/A	N/A	46708	N/A	N/A	N/A	N/A	N/A	N/A	1445
											
**117184 IL-10**											
1:10	40311	N/A	N/A	N/A	119338	N/A	N/A	180	N/A	1813	N/A
1:10	40401	N/A	230	N/A	72096	N/A	N/A	N/A	N/A	3621	318
1:10	40401	N/A	226	N/A	95800	N/A	N/A	N/A	N/A	3891	389
1:50	40311	N/A	N/A	N/A	95462	N/A	N/A	N/A	N/A	3836	N/A
1:50	40401	N/A	340	N/A	39419	N/A	N/A	N/A	N/A	4488	1855
1:50	40401	N/A	179	N/A	30223	N/A	N/A	N/A	N/A	4053	1308
											
**117187GM-CSF**											
1:10	40401	N/A	N/A	N/A	N/A	N/A	373	75824	N/A	N/A	875
1:10	40401	N/A	N/A	N/A	N/A	N/A	272	70453	N/A	N/A	721
1:10	40311	N/A	N/A	N/A	N/A	N/A	N/A	47332	N/A	N/A	N/A
1:10	40311	N/A	N/A	N/A	N/A	N/A	N/A	78882	N/A	N/A	769
1:50	40311	N/A	N/A	N/A	N/A	N/A	N/A	59603	N/A	N/A	N/A
1:50	40311	N/A	N/A	N/A	N/A	N/A	N/A	76623	N/A	N/A	1063
1:50	40311	N/A	N/A	N/A	N/A	N/A	N/A	19209	N/A	N/A	1193

The apparent cross-reactivity seen for MCP-1 and MIG might be caused by a medium additive present in the AIM V medium (a serum-free lymphocyte culture medium) used in a dilution step for these proteins. We tested several commonly used culture medias (AIM V, RPM1640, Iscoves and CellGenix DC media) in a 30-plex Luminex assay which also included a repeat test of the WHO standards. The results did identify low levels (3-62 pg/mL) of several analytes in the culture medias (HGF, FGF basic, RANTES, IL-17 and IL2R) but not MCP-1 or MIG (data not shown). The MCP-1 was again detected in the IL-8 and GM-CSF WHO standards and MIG in the IL-10 standard (as well as HGF, FGF basic and RANTES). We are investigating other possible sources of low levels of other cytokines and growth factors in the WHO standards.

**Figure 4:**
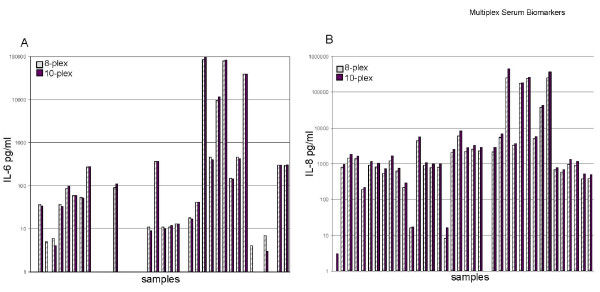
**Two plates run together compared for A) IL-6 and B) IL-8 values**. A set of 38 cell culture samples were run on both an 8-plex and a 10-plex plate. The values for IL-6 and IL-8 are compared on a log scale. Each plate had a unique upper limit. The values for IL-6 show excellent concordance, and the 8-plex upper limit was 1.8 times the upper limit. The IL-8 values were reproducibly higher (1.25×) in the 10-plex plate where the upper limit was 2.4 times higher.

As a test of the day-to-day reproducibility of two of the cytokines of particular interest, IL-6 and IL-8, a set of samples and controls were run in two different custom kits one day apart (with samples kept thawed, at 4°C overnight), in which both IL-6 and IL-8 were included in both kits. Notably, these two kits also had different standard curves and upper limits of detection. For IL-6, the 10-plex kit upper limit was 7, 400 pg/mL, while in the 8-plex, it was 13, 800 pg/mL (1.8 fold higher). For IL-8, the 10-plex upper limit was 24, 800 pg/mL and in the 8-plex, 10, 160 pg/mL (2.4 fold lower). When the values for the 38 samples were compared between the two kits, the ratio of the IL-6 values was 1.0 (median & mean), showing excellent concordance. For IL-8, where the upper limits were more disparate, the ratio of the values was 0.80, which was a small but significant difference (Figures 4A and 4B). These data indicate that the assay with the higher upper limit has larger measured values.

### Upper limit problem

**Figure 5:**
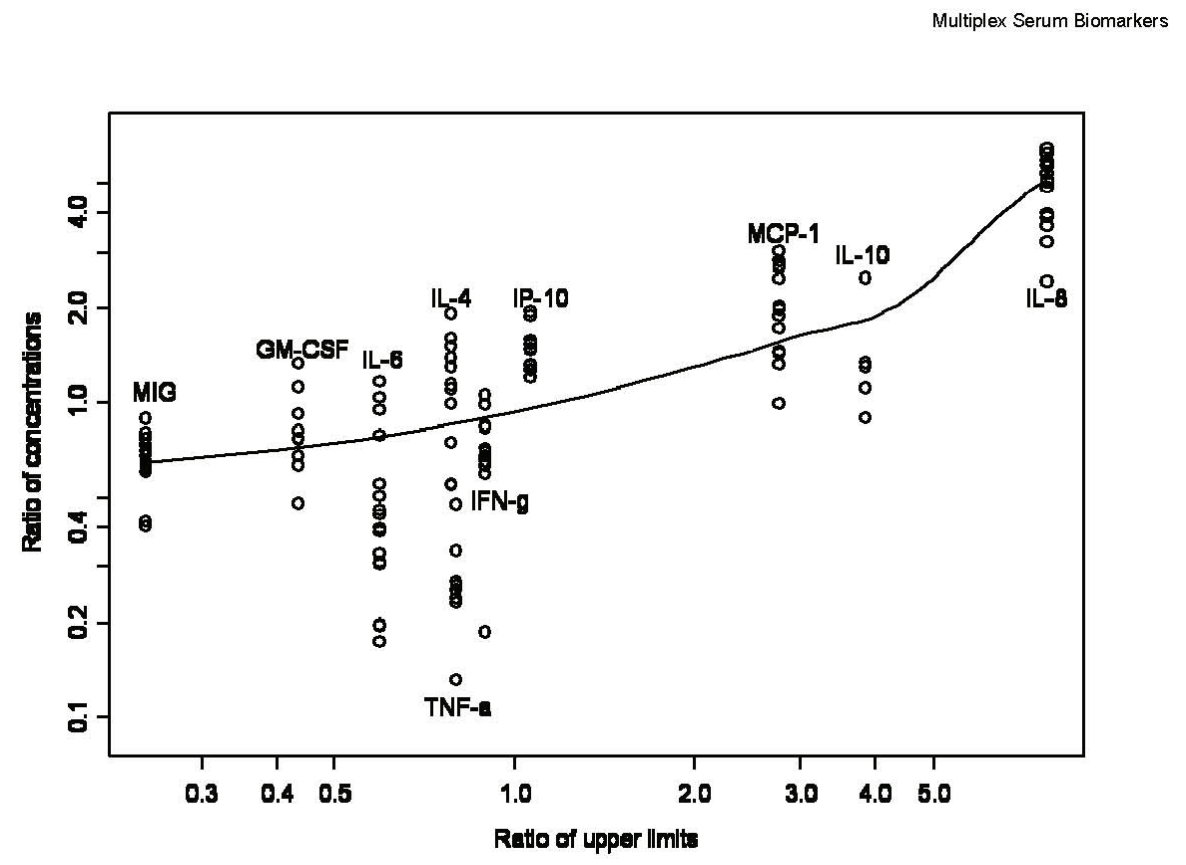
**Scatter plot of the late-to-early ratio of analyte concentrations versus the late-to-early ratio of assay upper limits of quantitation; a smooth curve is superimposed**. Early assays were done on 10/31/2005, 11/01/2005 or 2/17/2006; late assays were done on 5/13/2010.

The Luminex kits that we used at the different time points were not identical. In particular, we noticed that the upper limits of quantitation for individual analytes changed over time for the different kits. In principal, this should not affect the measured concentrations, because the kits include kit-specific standards to generate 8-point standard curves matched to the expected detection range. However, if the concentration determinations were affected, that would confound our interpretation of the observed changes in analyte concentration over time, and therefore we investigated that possibility. Data from assays done on 5/13/2010 ("late" assay) were compared to data from assays on 10/31/2005, 11/1/2005 or 2/17/2006 ("early" assays). Kits used in 2005 and 2006 had the same upper limits, and because no samples had assays done on the same date, results were combined. Figure 5 is a scatter plot of the late-to-early ratio of analyte concentrations versus the late-to-early ratio of assay upper limits assays with a smooth curve is superimposed. The late-to-early ratio of upper limits was different for each of the 10 analytes. Typically, 12 samples were assessed for each analyte. The correlation of the two ratios is highly significant (p < 10^-15^, Spearman's test). Therefore, we are concerned that assays performed at different times with different kits may not be comparable.

In this report, we detail reproducibility problems we encountered testing circulating cytokines, chemokines and growth factors by Luminex in serum samples which were stored over months to years under highly controlled conditions. Some of these changes were very dramatic: IL-8 increased 4-6 fold in old patient samples; MCP-1 decreased 4-6 fold in new patient samples, and up to 10-fold in healthy donor samples; IL-10 changed from negative to positive or positive to negative within the same old patient serum dataset (Figure 1). Our initial hypothesis was that the changes were entirely biological, and that despite standardized blood handling procedures and temperature-controlled freezer storage, some analytes became unstable over time or upon thaw. Two recent reports testing cytokine stability found most tested cytokines to be stable over 1-2 years at -80°C, and a subset (including IL-8 and IL-10) became unstable after 2-4 years [24,25]. Many of the proteins became unstable after repeated freeze-thaw cycles. If these were the only mechanisms, then the analytes we tested should have behaved consistently between our three datasets, because the change would be analyte-specific. This is not the only explanation, because, for example, MCP-1 increased over time in the majority of old patient samples and decreased over time in both HD and new patient sets.

Our study has a number of limitations. The more recently acquired HD and new patient data sets were tested within months of blood draw. A better analysis of the impact of storage time on analyte stability would require a large number of patients and HD samples stored for longer periods with costly repeated multiplex testing. We also limited the diversity of analytes we examined. Another variable was the time from blood draw to serum separation and freezing. Some of our samples were drawn within the laboratory and at our nearby clinic and processed within a few hours, while other old patient samples were shipped overnight and processed the following morning. However, the nature of these blood handling procedures reflects the unavoidable limitations inherent in transferring patient blood from the clinic to a central laboratory capable of standardized processing, as well as for multi-institutional trials where large numbers of patients can be treated and tested, but overnight shipping is required. Lastly, some of our healthy donor and control samples were run in duplicate, but to reduce costs, large numbers of patient sera were run in singlets. Due to the small average % CVs determined for many duplicates (Additional File 1, Table S1) this may have minimal impact on the trends we observed.

The Luminex assay has been shown (by ourselves [26] and others [27]) to show good correspondence to ELISA platform assays. In addition, the Luminex assay has good reproducibility from well-to-well, and from day-to-day (Figure 4). Also, our use of the R&D QC controls (Additional File 4, Table S4) indicate good reproducibility of recombinant analytes when mixed together. This may indicate that the serum matrix may impact reproducibility, and/or the biological impact of a tumor may lead to systemic changes (including altered glycosylation) which impact the assay.

This study also suggests that the changes in the upper limits of detection, which can vary substantially from kit to kit, month to month, and analyte to analyte from a single manufacturer, may impact the ability to determine analyte concentration. This impacts kit-to-kit reproducibility, and greatly increases the importance of comparing samples with the identical lot of kits with identical standard curve ranges. We attempted to dissect this further by requesting access to manufacturer QC data, but we were repeatedly denied access to any additional information specific to the testing performed on the kits we used.

We do not understand why the assay kit upper limits seem to affect assay performance in the systematic way that is evident in Figure 5. However, we have to conclude that the results of assays done with different kits cannot be directly compared. Therefore, the apparent changes in analyte levels over time that we observe may arise from the kit-to-kit variability: we cannot claim to observe changes in analyte levels over storage time at -80°C.

## Conclusions

In conclusion, the multiplex Luminex platform offers the opportunity to test a wide variety of analytes in the same sample, with minimal volume requirements, and good well-to-well and day-to-day reproducibility. These attributes are important when broadly searching for serum biomarkers. However, we find that a number of commonly tested candidate immunologic biomarkers show evidence of unexpected, large variability when tested retrospectively, after long storage times. This variability can be reduced by 1) performing assays with kits from a single lot, and potentially 2) minimizing storage time before retrospective analysis of banked serum.

## List of Abbreviations

IL: interleukin; TNF: tumor necrosis factor; GM-CSF: granulocyte-macrophage colony stimulating factor; PBMC: peripheral blood mononuclear cells; FDA: Food and Drug Administration; FBS: fetal bovine serum.

## Competing interests

The authors declare that they have no competing interests.

## Authors' contributions

LHB designed experiments, reviewed data, supervised assay conduct and wrote sections of the manuscript; DMP helped design experiments, designed and performed all statistical analyses and wrote sections of the manuscript; JMK provided many patient blood samples, reviewed data and wrote sections of the manuscript. All authors read and approved the final manuscript.

## Supplementary Material

Additional file 1 **Table S1: Luminex kit details**. This table includes upper and lower limits of detection and %CVs. Click here for file

Additional file 2 **Table S2: Healthy Donor Demographics**. This table includes age, race and gender information. Click here for file

Additional file 3 **Table S3: New Melanoma Patient Demographics**. This table includes age, race, gender and treatment information. Click here for file

Additional file 4 **Table S4: R&D Systems QC Control Data**. This table includes control sample values and %CVs. Click here for file
