# Supplementary material for: Multiplex serum biomarker assessments: technical and biostatistical issues
Source: J Transl Med. 2011 Oct 11;9:173. doi: 10.1186/1479-5876-9-173 (PMC3200183; doi:10.1186/1479-5876-9-173)
Supplement: Additional file 1 — Table S1: Luminex kit details. This table includes upper and lower limits of detection and %CVs. [file 1479-5876-9-173-S1.PDF]

New Supplementary Table 1. Luminex kit details

11-01-05 Assay Biosource Lot 205441 Expired 10/2006

	IL4[a]	IL6	IL8	IL10	TNFa	IFNg	GMCSF	IP10	MIG	MCP1
	pg/mL[b]	pg/mL	pg/mL	pg/mL	pg/mL	pg/mL	pg/mL	pg/mL	pg/mL	pg/mL
ULD[c]:	4600	6200	1600	4000	6500	14900	33500	1400	6900	8900
LLD[d]:	< 5	< 3	< 3	< 5	< 10	< 5	< 15	< 5	< 4	< 10
Standard curve % CV		(0.6-22)	(0-50)							(1.1-20)
(range) ave.[e]:		8.2	8.1							9.4

2-17-06 Assay Biosource Lot 205441 Expired 10/2006

	IL4	IL6	IL8	IL10	TNFa	IFNg	GMCSF	IP10	MIG	MCP1
	pg/mL	pg/mL	pg/mL	pg/mL	pg/mL	pg/mL	pg/mL	pg/mL	pg/mL	pg/mL
ULD:	4600	6200	1600	4000	6500	14900	33500	1400	6900	8900
LLD:	< 5	< 3	< 3	< 5	< 10	< 5	< 15	< 5	< 4	< 10
Standard curve % CV		(1.9-44)	(0.1-15)	(0.6-17)						(0-26)
(range) ave.:		8.6	5.8	6.1						8.2

2-2-10 Assay Invitrogen/Biosource Lot 746973A Expired 7/2011

	IL4	IL6	IL8	IL10	TNFa	IFNg	GMCSF	IP10	MIG	MCP1
	pg/mL	pg/mL	pg/mL	pg/mL	pg/mL	pg/mL	pg/mL	pg/mL	pg/mL	pg/mL
ULD:	3600	3700	12400	15400	5200	13300	14600	1490	1670	24600
LLD:	< 5	< 3	< 3	< 5	< 10	< 5	< 15	< 5	< 4	< 10
Standard curve % CV		(0-17)	(0-7)	(2-16)						(0-10)
(range) ave.:		6.3	2.3	5.5						2.3

5-13-10 Assay Invitrogen/Biosource Lot 750265A Expired 7/2011

	IL4	IL6	IL8	IL-10	TNFa	IFNg	GMCSF	IP10	MIG	MCP1
	pg/mL	pg/mL	pg/mL	pg/mL	pg/mL	pg/mL	pg/mL	pg/mL	pg/mL	pg/mL
ULD:	3600	3700	12400	15400	5200	13300	14600	1490	1670	24600
LLD:	< 5	< 3	< 3	< 5	< 10	< 5	< 15	< 5	< 4	< 10
Standard curve % CV		(0-9)	(0-11)	(0-13)						(0-12)
(range) ave.:		4.0	5.0	4.7						5.7

8-12-10 Assay Invitrogen/Biosource Lot 801830A Expired 11/2011

	IL4	IL6	IL8	IL-10	TNFa	IFNg	GMCSF	IP10	MIG	MCP1
	pg/mL	pg/mL	pg/mL	pg/mL	pg/mL	pg/mL	pg/mL	pg/mL	pg/mL	pg/mL
ULD:	3600	3700	12400	15400	5200	13300	14600	1490	1670	24600
LLD:	< 5	< 3	< 3	< 5	< 10	< 5	< 15	< 5	< 4	< 10
Standard curve % CV		(0-39)	(0-21)	(0-29)						(0-138)
(range) ave.:		7.9	4.1	8.4						22.5

[a] analyte
[b] units
[c] ULD, Upper limit of detection
[d] LLD, lower limit of detection (lowest value of the standard curve)
[e] For select analytes of particular interest, the range and average of duplicate well % CVs are shown for the 8-point standard curve and background wells.
