# Supplementary material for: Multiplex serum biomarker assessments: technical and biostatistical issues
Source: J Transl Med. 2011 Oct 11;9:173. doi: 10.1186/1479-5876-9-173 (PMC3200183; doi:10.1186/1479-5876-9-173)
Supplement: Additional file 2 — Table S2: Healthy Donor Demographics. This table includes age, race and gender information. [file 1479-5876-9-173-S2.PDF]

Supplementary Table 2. Healthy Donor Demographics

DRAW DATE	GENDER	AGE AT DRAW	RACE[1]
12/14/2009	Male	27	C
12/16/2009	Female	28	C
12/17/2009	Male	43	C
12/18/2009	Female	45	C
12/21/2009	Female	38	C
12/21/2009	Female	47	C
12/22/2009	Female	26	C
12/23/2009	Male	59	AA
12/24/2009	Female	56	C
12/28/2009	Female	47	C

[1] C = Caucasian, AA = African American
