# Supplementary material for: Multiplex serum biomarker assessments: technical and biostatistical issues
Source: J Transl Med. 2011 Oct 11;9:173. doi: 10.1186/1479-5876-9-173 (PMC3200183; doi:10.1186/1479-5876-9-173)
Supplement: Additional file 3 — Table S3: New Melanoma Patient Demographics. This table includes age, race, gender and treatment information. [file 1479-5876-9-173-S3.PDF]

Supplementary Table 3. New Melanoma Patient Demographics

PATIENT NUMBER	AGE AT DRAW	GENDER	RACE[1]	30 DAYS PRIOR TREATMENT[2]
1	60	FEMALE	C	NONE
2	44	MALE	C	
3	74	FEMALE	C	HD Interferon alpha-2b subcutaneous phase w/ significant toxicity Oral Temodarprior, but on hold for 2-3 weeks
4	81	MALE	C	Synthroid 112 mcg, Nexium
5	55	MALE	C	NONE

[1] C = Caucasian

[2] HD = High dose
