# Supplementary material for: Multiplex serum biomarker assessments: technical and biostatistical issues
Source: J Transl Med. 2011 Oct 11;9:173. doi: 10.1186/1479-5876-9-173 (PMC3200183; doi:10.1186/1479-5876-9-173)
Supplement: Additional file 4 — Table S4: R&D Systems QC Control Data. This table includes control sample values and %CVs. [file 1479-5876-9-173-S4.PDF]

Supplementary Table 4. R&D Systems QC Control Data

Description	Assay Date	IL-4 pg/mL	IL-6 pg/mL	IL-8 pg/mL	IL-10 pg/mL	TNF-α pg/mL	IFN-g pg/mL	GM-CSF pg/mL	IP-10 pg/mL	MIG pg/mL	MCP-1 pg/mL
130479 Multiplex Control, low	2/2/2010	154	109	167	28	***	60	29	***	***	76
**QC02 Control, Low**	**2/2/2010**	36	105	66	19	75	40	37	N/A	N/A	29
131216 Multiplex Control, low	5/13/2010	149	88	146	31	***	60	21	***	***	72
131216 Multiplex Control, low	8/11/2010	131	88	126	27	***	56	23	***	***	48
**QC02 Control, low**	**5/10 to 8/10**	39	109	71	21	75	36	39	N/A	N/A	31
% CV[1]		12%	15%	18%	7%	N/A	5%	20%	N/A	N/A	25%
130480 Multiplex Control, med	2/2/2010	1349	582	1412	284	27	605	242	***	***	731
**QC02 Control, med**	**2/2/2010**	345	780	566	206	712	346	362	N/A	N/A	286
131217 Multiplex Control, med	5/13/2010	1413	513	1200	280	56	580	239	***	20	736
131217 Multiplex Control, med	8/11/2010	1102	556	1129	282	55	597	231	***	48	477
**QC02 Control, med**	**5/10 to 8/10**	361	800	614	214	735	330	378	N/A	N/A	303
% CV[1]		13%	7%	16%	2%	34%	1%	4%	N/A	N/A	24%
130481 Multiplex Control, high	2/2/2010	5655	1878	4928	1182	227	2444	926	***	39	2746
**QC02 Control, high**	**2/2/2010**	1258	2891	2018	817	2828	1300	1396	N/A	N/A	1245
131218 Multiplex Control, high	5/13/2010	5776	1642	3961	1035	773	2252	802	***	53	2358
131218 Multiplex, Control, high	8/11/2010	OOR>	1706	4001	1073	726	2133	936	4	125	1791
**QC02 Control, high**	**5/10 to 8/10**	1285	2813	2056	822	2802	1175	1418	N/A	N/A	1209
% CV[1]		N/A	5%	13%	7%	52%	2%	8%	N/A	N/A	19%

[1] % CV = % coefficient of variation
